# Preparation and Properties of Nanoparticles of Calcium Phosphates With Various Ca/P Ratios

**DOI:** 10.6028/jres.115.018

**Published:** 2010-08-01

**Authors:** Limin Sun, Laurence C. Chow, Stanislav A. Frukhtbeyn, John E. Bonevich

**Affiliations:** American Dental Association Foundation Paffenbarger Research Center, Polymers Division, National Institute of Standards and Technology, Gaithersburg, MD 20899-8546, U.S.A.; Metallurgy Division, National Institute of Standards and Technology, Gaithersburg, MD 20899-8555, U.S.A.

**Keywords:** calcium phosphate, Ca/P ratio, ion activity products (IAP), nanoparticle, solubility, spray drying

## Abstract

This study aimed at preparing and studying the properties of nanoparticles of calcium phosphate (nCaP) with Ca/P ratios ranging from 1.0 to 1.67 using a spray-drying technique. Micro-structural analyses suggested that the nCaPs with Ca/P ratios of 1.67 to 1.33 were nano-sized amorphous calcium phosphate (ACP) containing varying amounts of acid phosphate and carbonate. The nCaP with Ca/P ratio of 1 contained only nano-sized low crystalline dicalcium phosphate (DCP). BET measurements of the nCaPs showed specific surface areas of (12 ± 2 to 50 ± 1) m^2^/g, corresponding to estimated equivalent spherical diameters of (38 to 172) nm. However, dynamic light scattering measurements revealed much larger particles of (380 ± 49 to 768 ± 111) nm, owing to agglomeration of the smaller primary nano particles as revealed by Scanning Electron Microscopy (SEM). Thermodynamic solubility measurements showed that the nCaPs with Ca/P ratio of 1.33 – 1.67 all have similar solubility behavior. The materials were more soluble than the crystalline hydroxyapatite (HA) at pH greater than about 4.7, and more soluble than β-tricalcium phosphate (β-TCP), octacalcium phosphate (OCP) and DCP at pH above 5.5. Their solubility approached that of α-tricalcium phosphate (α-TCP) at about pH 7. These nCaPs, which cannot be readily prepared by other currently available methods for nanoparticle preparation, have potential biomedical applications.

## 1. Introduction

Calcium phosphates are of interest for many biomedical applications due to their good biocompatibility and bioactivity. Hydroxyapatite (HA) has been used as implant coatings [1] and bone substitutes [2]. Amorphous calcium phosphate (ACP) are used as remineralization agents both in-situ [3] and in tooth restorative materials [4]. Dicalcium phosphate anhydrous (DCPA) and dicalcium phosphate dihydrate (DCPD) [5], octacalcium phosphate (OCP) [6] and other calcium phosphate compounds [5, 7–9] are used either as components or formed as products of calcium phosphate bone cements. Previous studies on nano calcium phosphates have focused almost exclusively on nano HA, primarily because it is considered as a prototype of bioapatites, which are in nano crystalline forms [10]. Most of these preparations were done in solution environment, such as chemical precipitation [11–12], sol-gel [13–14], microemulsion [15–16], electrodeposition [17], and mechanochemical preparation followed by hydrothermal treatment [18]. These methods generally can be used for preparing nano HA only because HA is the least soluble calcium phosphate under most solution conditions; hence is the phase that would form exclusively. Nanoparticles of the more soluble calcium phosphate phases, such as monocalcium phosphate monohydrate (MCPM), DCPA, DCPD, OCP, ACP, have not been prepared by these methods.

Previously, we have successfully prepared nano forms of HA, DCPA and MCPM using a spray drying method with one-liquid nozzle [19–21]. The present study was aimed at preparing and studying the properties of nano forms of additional calcium phosphates of molar Ca/P ratios from 1.0 to 1.67, corresponding to the ratios of crystalline DCPA to HA.

## 2. Materials and Methods

### 2.1 Preparation of nCaP Using the Spraying Drying Method

**Fig. 1:**
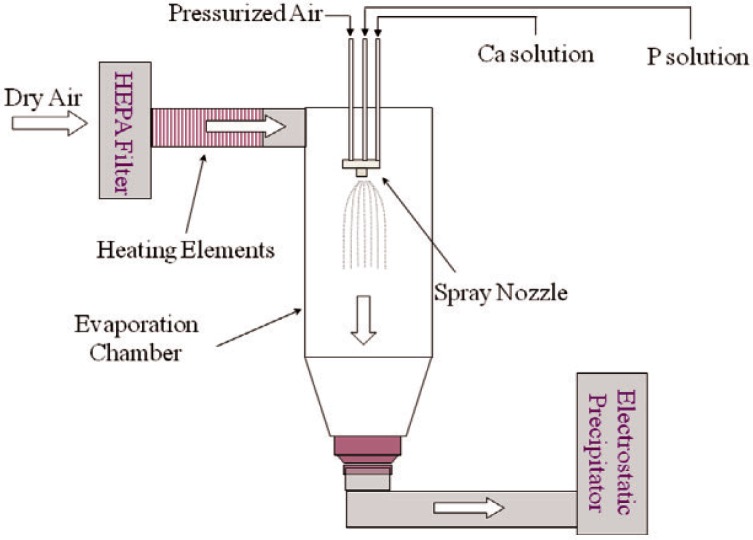
Schematic drawing of the spray drying apparatus.

The preparation method, which employed a spray-drying technique with the use of a two-liquid nozzle, was modified from a previous approach using a one-liquid nozzle [19–21] and similar to the method used for preparing nano calcium fluoride [22]. Specifically, a dilute calcium hydroxide solution (≈ 4 mmol/L) and a dilute phosphoric acid solution (with a concentration corresponding to a desired Ca/P molar ratio) were simultaneously fed to the nozzle and atomized into a stream of heated air flowing through an evaporation chamber (Fig. 1). Evaporation of the water from the micrometer-sized droplets of the combined solution led to nucleation of the compound to be prepared following Eqs. (1 or 2), forming either an acidic or a basic calcium phosphate (the amount of water released would be reduced if a hydrated salt is formed). At the end of the evaporation chamber, the nano particles were trapped by an electrostatic precipitator (MistBuster, Air quality Engineering, Inc., Minneapolis, MN)^1^ and collected at the end of the process. The water vapor was removed with the air flow.

(1) $$\begin{array}{c} x\;\text{Ca}{\left(\text{OH}\right)}_{2}+y\;H_{3}{\text{PO}}_{4}\to {\text{Ca}}_{x}H_{3y-2x}{\left({\text{PO}}_{4}\right)}_{y}\\ +2x\;H_{2}O↑ \end{array}$$

when x ≤ 1.5y

(2) $$\begin{array}{c} x\;\text{Ca}{\left(\text{OH}\right)}_{2}+y\;H_{3}{\text{PO}}_{4}\to {\text{Ca}}_{x}{\left({\text{PO}}_{4}\right)}_{y}{\left(\text{OH}\right)}_{2x-3y}\\ +3y\;H_{2}O↑ \end{array}$$

when x ≥ 1.5y

where x/y is the Ca/P molar ratio of the compound formed. Four Ca/P ratios were used in this study: 1.67, 1.5, 1.33 and 1.0, which corresponded to the Ca/P ratios of HA, ACP/TCP (tri-calcium phosphate), OCP and DCPA/DCPD, respectively. The parameters used in spray-drying process, which can affect the chemistry and particle size of the products, were inlet (≈ 85 °C) and outlet (≈ 50 °C) temperatures of the spraying chamber, liquid feeding rate (10 ml/min for each solution), the orifice size of the spray nozzle (0.381 mm) and atomization air pressure (276 kPa or 40 psi). These parameters were maintained unchanged in the current study.

### 2.2 Characterization of nCaP

#### 2.2.1

Powder X-ray Diffraction (XRD) (DMAX 2200, Rigaku Denki Co., Ltd., The Woodlands, TX) was used to identify the crystalline phases [23] present in the product. Scans were performed over a 2θ range of 10° to 60° at rate of 1°/min with a sampling interval of 0.01°, which corresponds to the estimated standard uncertainty.

#### 2.2.2

A NEXUS 670 FT-IR spectrometer (Thermo Nicolet, Madison, WI) was used to record the infrared spectra of the nano powders. The powders were mixed well with IR quality KBr at a mass ratio of ≈ 1:400 and the mixture was then pressed into a pellet in a 13 mm diameter evacuated die. The absorbance spectra were acquired over the range of 400 cm^‒1^ to 4000 cm^‒1^ using a DTGS detector and KBr beam splitter, with a resolution of 2 cm^‒1^. Each spectrum was scanned 32 times to increase the signal-to-noise ratio. The estimated standard uncertainty of wavelength was ± 4 cm^‒1^.

#### 2.2.3

The particle morphology and microstructure of nCaP were examined using Scanning Electron Microscopy (SEM, JSM-5300, JEOL, Peabody, MA) and Transmission Electron Microscopy (3010 HREM TEM, JEOL). The TEM sample was prepared by depositing particles onto a holey carbon film-coated copper grid from a well-sonicated dilute suspension in acetone to minimize agglomeration.

#### 2.2.4

Multipoint BET (Brunauer, Emmett, and Teller) surface area analyses were done (Gemini 2375 Surface Area Analyzer, Micromeritics, Norcross, GA) with ultra high purity nitrogen as the adsorbate gas and liquid nitrogen as the cryogen. All samples were pretreated with degassing at 90 °C for 1 h followed by 105 °C overnight with ultra high purity nitrogen purge before the measurement. Analyses were conducted on replicate samples to establish standard deviation. In this and other measurements in the present study, the standard deviation was taken as the standard uncertainty. The particle size of the primary crystals of nCaP was estimated from the BET surface area by calculating equivalent spherical diamater, or BET particle diameter (d_BET_), from the fundamental equation: d_BET_ = 6/(ρ · S_w_), where ρ is the density of the powder and S_w_ is the specific surface area [24]. The theoretical densities of stoichiometric HA, ACP, OCP and DCPA, which were (3.156, 2.895, 2.61 and 2.89) g / cm^3^, respectively [25], were used for the calculations of the nCaP with Ca/P ratios of 1.67, 1.5, 1.33 and 1.

#### 2.2.5

The particle size distribution of the nCaP powders were determined by dynamic light scattering (DLS) (90 Plus particle sizer, Brookhaven Instruments, Holtsville, NY) at a wavelength of 532 nm at 25 °C. Samples were prepared by dispersing small amounts of powder in 100 % ethanol followed by sonication for 10 min. A viscocity of 1.19 cP and refractive index of 1.36 were used for the calculation.

#### 2.2.6

A thermo gravimetric analyzer (TGA) (Q500, TA Instruments, New Castle, DE) was used to determine the mass loss of the nano powder samples as a function of increasing temperature. The samples were heated up to 950 °C at 10 °C/min in a flowing air (20 ml/min). Estimated standard uncertainty of temperature calibration was ± 5 °C.

#### 2.2.7

The solubility properties of the nCaP were assessed by equilibrating the material in a 0.92 mmol/L phosphoric acid (H_3_PO_4_) solution [19] at 37 °C under a nitrogen atmosphere. The solution also contained 150 mmol/L potassium chloride (KCl) as an electrolyte background, and 1 mmol/L magnesium chloride (MgCl_2_) and 0.04 mmol/L sodium pyrophosphate (Na_4_P_2_O_7_) to inhibit the precipitation of crystalline HA. A 100 mg sample of the nCaP powder was added to 100 mL of the solution under constant stirring (30,000 Rad/s or 500 rpm). The powder was allowed to dissolve until a stable pH was achieved, and a 2 mL aliqout of slurry was removed and filtered for Ca and P analyses using spectrophotometry methods [26]. Additional H_3_PO_4_ was then added to the solution, leading to more sample dissolution and a new equilibration pH at a more acidic value. A 2 mL aliquot of slurry was again taken for calcium and phosphate analyses. This process was repeated several times to produce conditions equivalent to dissoving the nCaP samples in solutions with initial H_3_PO_4_ concentrations of (0.92, 1.83, 2.98, 5.11, 6.74, 8.41, and 9.55) mmol/L. The pH, calcium ([Ca]), and phosphate ([P]) concentration values were used to calculate solution ion activity products (IAP) with respect to HA, TCP/ACP, OCP, and DCPA/DCPD Eqs. (3 to 6), respectively) using the software ‘Chemist’ (Micromath, Saint Louis, MO).

(3) $$\text{IAP}(\text{HA})={\left({\text{Ca}}^{2+}\right)}^{10}{\left({\text{PO}}_{4}^{3-}\right)}^{6}{\left({\text{OH}}^{-}\right)}^{2}$$

(4) $$\text{IAP}(\text{TCP}/\text{ACP})={\left({\text{Ca}}^{2+}\right)}^{3}{\left({\text{PO}}_{4}^{3-}\right)}^{2}$$

(5) $$\text{IAP}(\text{OCP})={\left({\text{Ca}}^{2+}\right)}^{8}{\left({\text{PO}}_{4}^{3-}\right)}^{6}{\left(H^{+}\right)}^{2}$$

(6) $$\text{IAP}(\text{DCPA})=\left({\text{Ca}}^{2+}\right)\left({\text{HPO}}_{4}^{3-}\right)$$

where quantities in () on the right hand side of the equations denote ion activities. Solubility measurements were conducted in replicate to establish standard deviations.

#### 2.2.8

One-way ANOVA (analysis of variance) was used to analyze the data obtained from the BET, DLS and solubility studies for the nCaP with various Ca/P ratios.

## 3. Results

**Fig. 2:**
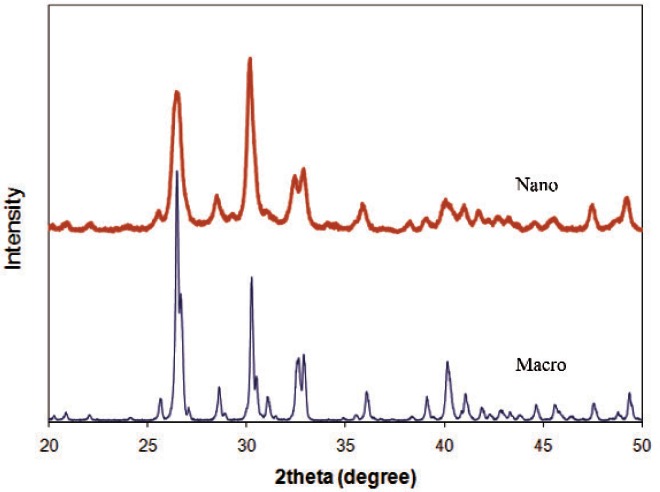
XRD pattern of nano and macro DCPA.

**Fig. 3:**
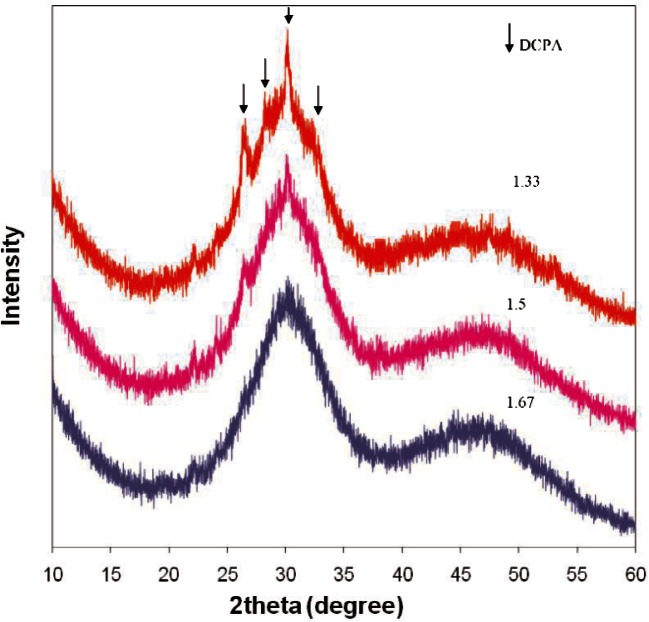
XRD pattern of nano calcium phosphates with Ca/P = 1.33 to 1.67.

**Fig. 4:**
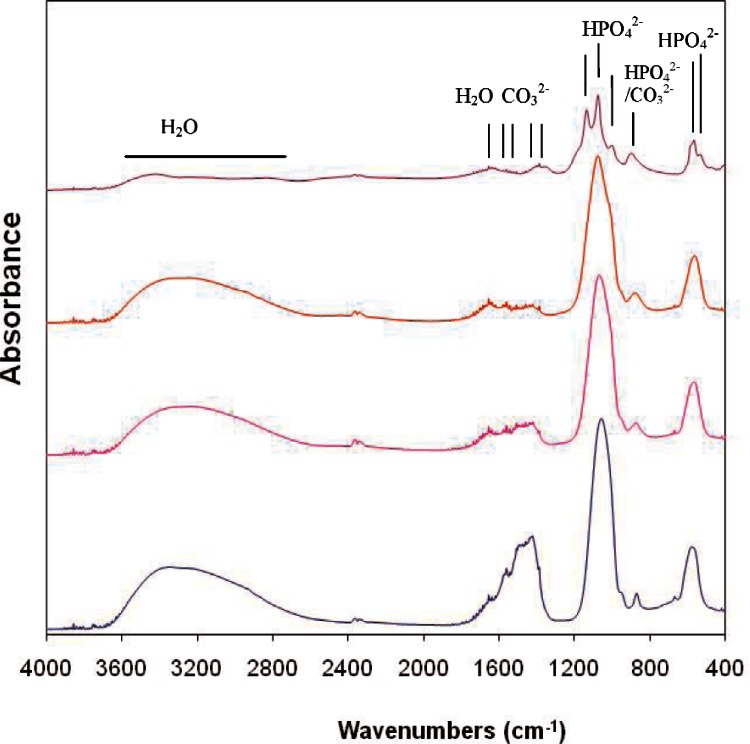
FTIR of nano calcium phosphates with Ca/P = 1 to 1.67.

XRD patterns of the Ca/P = 1 material (Fig. 2) showed it to be low crystalline DCPA with a minor amount of DCPD. Compared to its highly crystalline counterpart, the peaks of the nano DCPA were broader, indicating a finer crystal size and/or a less perfect structure. XRD patterns of the Ca/P = 1.33 – 1.67 materials (Fig. 3) displayed patterns similar to that reported for ACP [27]. Minor amounts of low crystalline DCPA are also present. FTIR of the Ca/P = 1 material (Fig. 4) displayed a typical DCPA spectrum with major acid phosphate bands at (534, 566, 887, 989, 1060 and 1120) cm^‒1^. FTIR of the Ca/P = 1.33 – 1.67 materials (Fig. 4) showed broad unresolved spectra similar to that of carbonated ACP, with phosphate bands at (573, 968 and 1030 to 1090) cm^‒1^, acid phosphate bands at (887 and 989) cm^‒1^, carbonate bands at (1390, 1440, 1510, 1580) cm^‒1^ and absorbed water bands at (1650 and 2700 to 3700) cm^‒1^. Lower Ca/P ratio materials contained less carbonate and more acid phosphate.

**Fig. 5:**
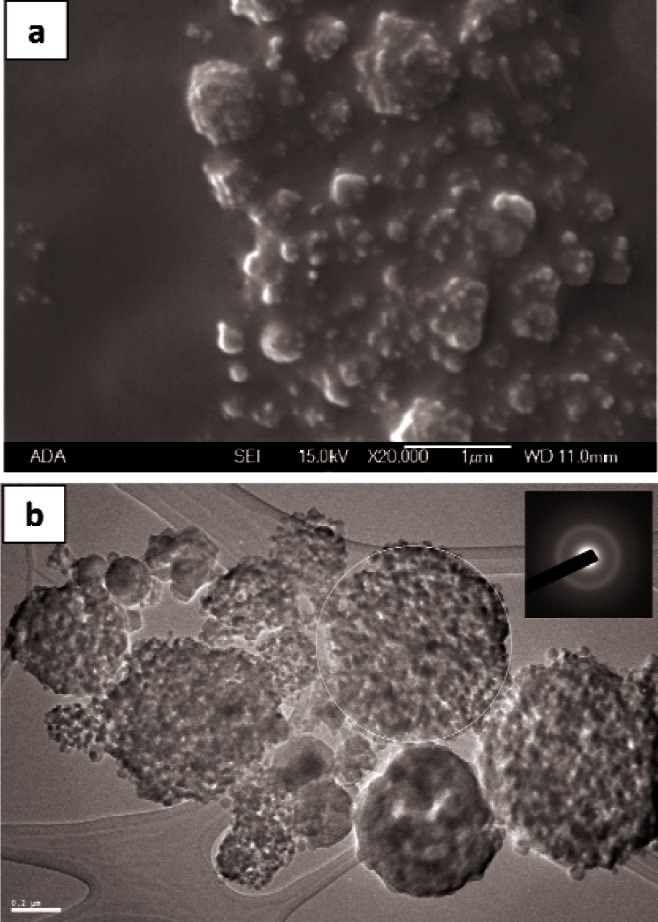
SEM and TEM of nano calcium phosphate powders (Ca/P = 1.67 sample).

SEM observations of the nCaP with Ca/P = 1.67 (Fig. 5a) showed presence of both nano particles of ~ 50 nm and large agglomerates of several hundred nanometers. The larger particles exhibited numerous spherical protuberances on the surfaces, suggesting that they were formed during the spray drying process through fusion of the much smaller primary nano particles. TEM (Fig. 5b) confirmed that the nano Ca-P particles contained clusters comprised of still finer particles of (10 to 15) nm in size, and indicated that the material has an amorphous structure.

**Fig. 6:**
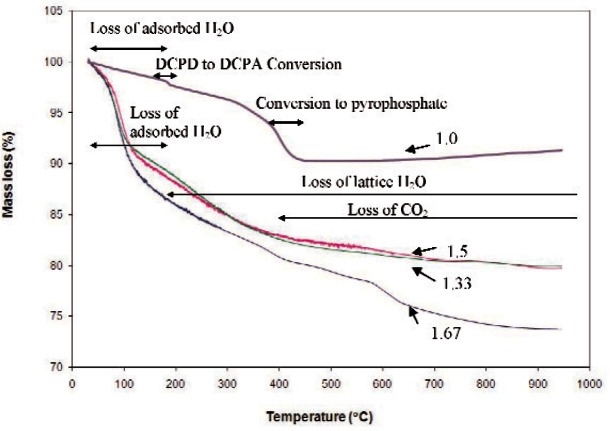
TGA of nano calcium phosphates with Ca/P = 1 to 1.67.

TGA of the Ca/P = 1 material (Fig. 6) showed a mass loss of ≈10 % up to around 400 °C. In addition to loss of adsorbed water, there were two characteristic losses at ≈200 °C and ≈400 °C, which were due to conversion of the minor amount of DCPD impurity phase to DCPA and conversion of DCPA to γ-calcium pyrophosphate, respectively [28]. TGA curves of the 1.33 to 1.67 materials (Fig. 6) showed that all samples had significant mass losses (17 % to 19 %) when heated up to 400 °C, which can be ascribed to the loss of adsorbed water followed by the lattice water due to pyrophosphate formation and/or the reaction, OH^‒^ + HPO_4_^‒2^ → PO_4_^‒3^ + H_2_O↑ [25]. In addition to the loss of water, the loss of CO_2_ starting from around 400 °C and up could contribute to the total weight loss in the Ca/P = 1.67 sample [28].

The BET specific surface areas of the nCaP powders ranged between (12 ± 2 and 50 ± 1) m^2^/g (Table 1); the values were smaller for the powders with lower Ca/P ratios. These values corresponded to an estimated equivalent spherical diameter (d_BET_) of (38 to 172) nm. The particle size distributions of the powders measured by DLS (d_DLS_) were usually bimodal. The Ca/P = 1.33 to 1.67 materials exhibited one major peak at (100 to 200) nm and one minor peak at (300 to 600) nm, whereas the Ca/P = 1 material exhibited one major peak at (500 to 950) nm and one minor peak at (2 to 4) μm. The mean particle size from DLS ranged between (380 to 768) nm, which strongly suggested the presence of agglomerates of the smaller nano-sized primary particles. These results are in good agreement with SEM and TEM observation (Fig. 5).

**Fig. 7:**
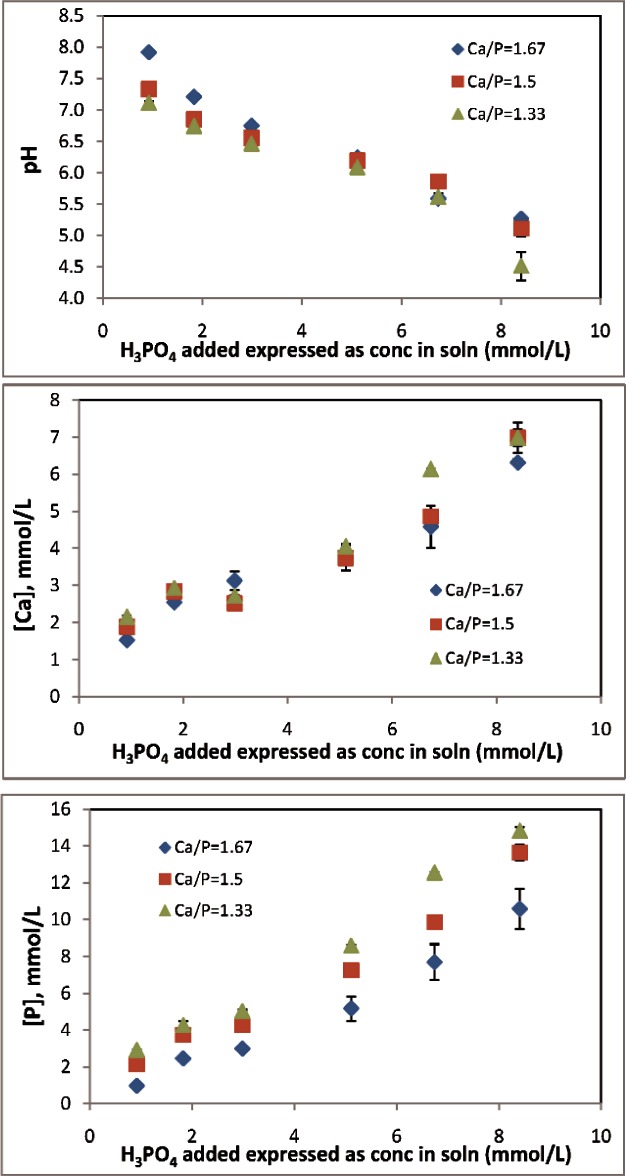
Dissolution curves of nano calcium phosphates with Ca/P = 1.33 to 1.67.

No reliable solubility data were obtained for the nCaP with Ca/P = 1 because of its extremely high solubility, which led to rapid precipitation of other less soluble phases in a preliminary study. For the other 3 nCaP materials with Ca/P = 1.33 to 1.67, the pH of the equilibrating suspension decreased with each addition of H_3_PO_4_ as expected (Fig 7a). For a given amount of H_3_PO_4_ addition, the pH was the highest for the solution equilibrated with the Ca/P = 1.67 material and the lowest with the Ca/P = 1.33 material. This confirmed that the material with a higher Ca/P ratio had a greater basicity. The [Ca] of the equilibrating solutions increased with increasing amount of H_3_PO_4_ addition. The [Ca] vs. pH profiles for the 3 nCaP materials did not separate, i.e., they fell on one general line (Fig. 7b). In contrast, the [P] was the highest for the Ca/P = 1.33 material and lowest for the Ca/P = 1.67 material (Fig. 7c).

**Fig. 8:**
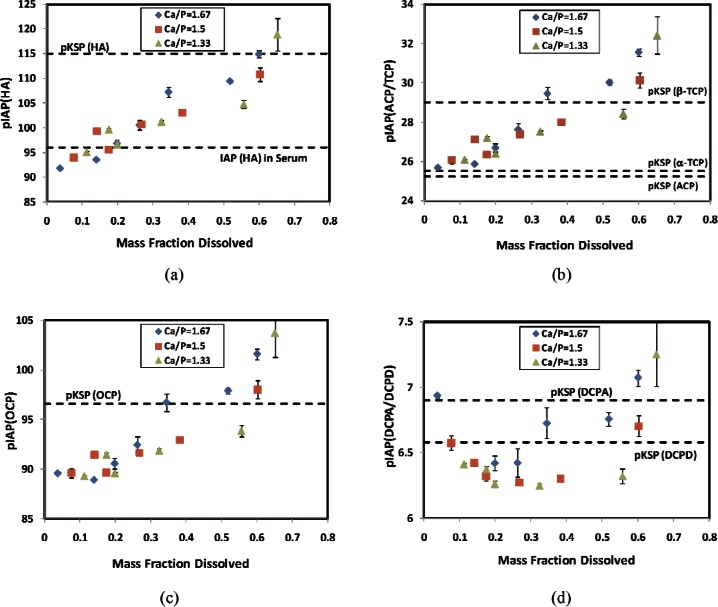
pIAP of the solution equilibrated with nano calcium phosphates with Ca/P = 1.33 to 1.67 under different pHs.

The pIAP values with respect to the relevant calcium phosphate phases (HA, ACP/TCP, OCP, and DCPA) of each solution equilibrated with a nCaP sample were calculated from the pH, [Ca] and [P] values, and an estimated ionic strength of the solution. Since the solubility data points were obtained by sequential additions of H_3_PO_4_ to the equilibrating suspension (Sec. 2.2.7), a progressively greater fraction of the nCaP sample would have dissolved after each addition of H_3_PO_4_. In Fig. 8, the calculated pIAP values are plotted as a function of the mass fraction of the sample dissolved, which was estimated from the measured [Ca] of the equilibrating solution. The pIAP profiles of the 3 nCaP materials did not show clear separation, suggesting that the 3 materials do not have distinctly different thermodynamic solubility properties. Figure 8a shows that the most soluble fractions (about 10 % mass fraction) of all 3 nCaPs had very high solubility such that they are soluble in serum, which is supersaturated with respect to HA. The solubility decreased with greater fraction of the sample dissolved and it approached the solubility of HA after about 60 % mass fraction of the sample had dissolved. It should be noted that because HA is the most stable phase in the entire pH range of the solubility experiments (pH from approximately 8 to 4.5), it is HA and not the other CaP phases that dictates the solid-solution equilibrium in the experiments. Thus the pIAP values plotted in Figs. 8b, 8c and 8d depict the saturation levels with respect to ACT/TCP, OCP, and DCPA, respectively, of the solutions that were in equilibrium with a HA phase. It can be seen that the solutions were supersaturated with respect to ACP/TCP and OCP until about 60 % of the sample had dissolved. Surprisingly, the solutions were also supersaturated with respect to DCPA (Fig. 8d) nearly independent of the fraction of the sample dissolved.

**Fig. 9:**
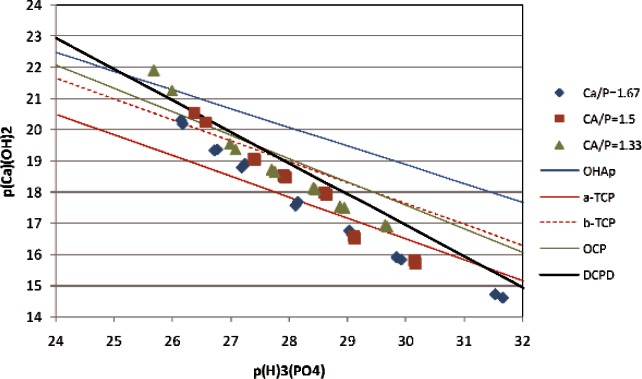
Potential phase diagram showing solubility isotherms of nCaP and the related calcium phosphate salts.

The solubility data can also be presented in the form of a potential diagram (Fig. 9) [29]. The straight lines denote the solubility isotherms of the well crystallized phases, HA, α-TCP, β-TCP, OCP, and DCPD. In this diagram, for a given p[(Ca^2+^)(OH^‒^)^2^] level, the solution pH increases with increasing p[(H^+^)^3^(PO_4_^3–^)] level, and for a given p[(H^+^)_3_(PO_4_^3–^)] level, the pH decreases with increasing p [Ca^2+^)(OH^‒^)^2^] level. Further, points to the lower left of an isotherm represent solution compositions that are supersaturated with respect to the solid. Thus, the potential diagram showed that the equilibrating compositions of all three nCaP materials form a general group, and the solutions were mostly supersaturated with respect to HA, OCP and β-TCP and under-saturated with respect to α-TCP, as described above. However, it can be seen from the diagram that the solubility points fit the DCPD isotherm better than any other crystalline CaP phases, suggesting dissolution properties of the nCaP materials may best be described as similar to that of DCPD (or DCPA). The situation is also reflected in Fig. 8d, in which the pIAP (DCPA/DCPD) values remain nearly constant with pH (except at the higher and lower ends of the curve), whereas the pIAP values with respect to other CaP phases vary significantly with pH (Figs. 8a, 8b, and 8c).

## 4. Discussion

As mentioned before, most of the conventional as well as nano forms of calcium phosphate compounds were prepared in a solution environment [25]. Formation of the mineral particles occurs through nucleation and crystal growth processes during which the solution composition remains essentially unchanged or changed slowly. In the two-liquid spray drying process, the rapid water evaporation of the mixed Ca and P-containing solution facilitated by the hot air flow also results in the nucleation of the calcium phosphate products. However, in this process, as the water in a droplet is evaporated rapidly, the solution composition would change from an undersaturated or mildly supersaturated condition to highly supersaturated condition within seconds. This would prevent and strongly limit the newly formed nuclei or crystallites to grow further. The contrast between the two types of preparation methods is especially apparent for those calcium phosphate phases with higher solubilities, such as DCPA and DCPD [25]. Because crystals of these salts would form in a more concentrated solution, they usually can grow to several to hundreds microns in size in the conventional solution preparation process. In contrast, the primary crystals of the nCaP with Ca/P = 1 prepared by spray drying process were much smaller (172 nm in Table 1).

The rapid solid formation in the spray drying process could also result in an imperfect crystal structure, especially for those phases with more complex structures, such as HA and OCP. This may explain why the XRD patterns of all nCaP materials (Fig. 3), except the one with Ca/P = 1 which has a DCPA crystal structure (Fig. 2), were in the form of a broad hump, characteristics of ACP. However, some acid phosphate was present in the nCaP with Ca/P ratio of 1.5 and 1.67, suggesting that the material bears some resemblance to Ca-deficient apatitic materials. The decreased primary particle size observed for the nCaPs with higher Ca/P ratio (Table 1) suggests that the acid phosphate-containing CaP could grow larger during spray drying formation. The presence of carbonate in the materials undoubtedly came from atmospheric CO_2_. The presence of less carbonate in the acid phosphate-containing nCaP with lower Ca/P ratios (Fig. 4) could be owing to the lower solubility of the atmospheric CO_2_ in the lower pH reactant solutions.

Under similar preparation and drying conditions, the nCaPs with Ca/P = 1.33 to 1.67 appeared to contain 8 % of its mass as adsorbed water (mass loss when heated to 100 °C), whereas the Ca/P = 1 material contained only slightly more than 1% adsorbed water (Fig. 5). This observation is in good agreement with the results of IR measurements (Fig. 4). Since the BET areas of the higher Ca/P materials were 3 to 4 times that of the Ca/P = 1 material (Table 1), affinity for moisture seems to be related to the surface areas/particle size of the material. It is possible that the water contents in the materials may be affected by adjusting the heating efficiency of the spray drying process or reduced using a post-heat treatment.

The nCaP powders with Ca/P = 1.33 to 1.67 showed similar dissolution characteristics (Figs. 8 and 9). This may be explained by the fact that the thermodynamic solubility is primarily a function of the free energy of the solid phase, which, in turn, is a function of the crystal structure rather than the Ca/P ratio. Since all three nCaP materials have an amorphous structure, they should have similar free energies, leading to similar thermodynamic solubility behavior. It is not entirely clear why the nCaP solubility can best described by DCPD’s dissolution properties as seen from the potential diagram (Fig. 9).

The small size and high solubility of the nCaP with various ratios made them good candidates for several applications. One example is that the nCaP powders could be used as the nano-fillers in dental resin composites to continuously release Ca and P ions into the oral environment, which would help prevent demineralization and promote remineralization [30]. Calcium phosphates have also been used as drug delivery carrier due to their excellent biological properties and high surface interaction properties [31–32]. The nCaP particles can be more advantageous in this respect as their small size and large surface can facilitate incorporation of drugs and easy transport in the physiological system. The dependence of solubility on pH and the fraction of material dissolved suggests the possibility of designing nCaP-based system with desired drug release profiles.

## 5. Summary

Nano-sized calcium phosphates with Ca/P ratios of 1.0 to 1.67 were prepared using a spray drying method. Micro-structural analyses results revealed the Ca/P = 1 materials to be DCP only, while Ca/P = 1.33 to 1.67 materials to be amorphous calcium phosphates containing varying amounts of acid phosphate and carbonate. The nCaPs with Ca/P ratio of 1.33 – 1.67 have similar thermodynamic solubility behavior; all exhibited a range of solubility expressed as IAP with respect to the relevant CaP phases. All of the nCaPs were more soluble than HA, β-TCP, OCP and DCPA until after approximately 60 % mass fraction of the material had dissolved. The highly soluble nCaP also have small particle size and large surface area, making them potentially useful for applications such as tooth remineralization and controlled drug release.

## Figures and Tables

**Table 1:** BET specific surface area and particle size of the nCaP powders

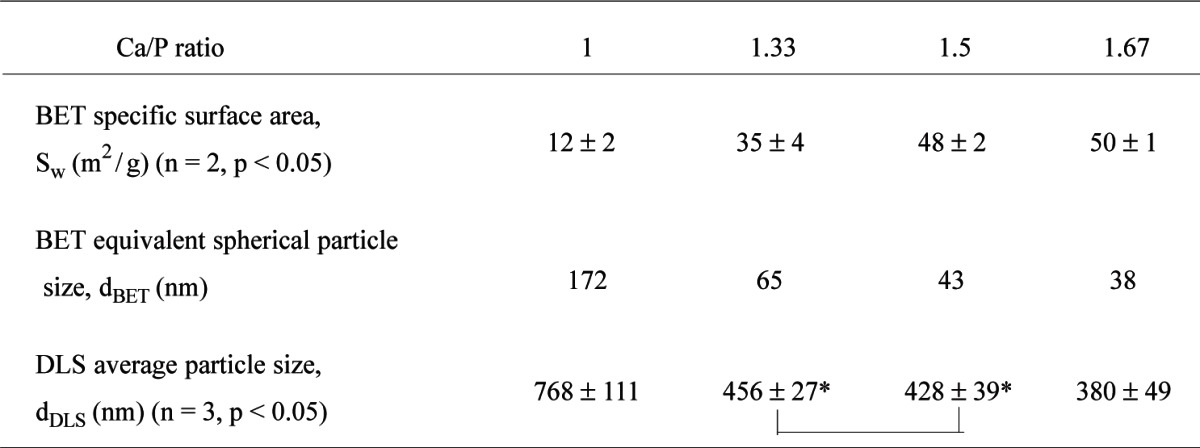

*The values connected are not significantly different from each other (p > 0.05)

## Footnotes

1 Certain commercial equipment, instruments, or materials are identified in this paper to specify the experimental procedure adequately. Such identification is not intended to imply recommendation or endorsement by the National Institute of Standards and Technology or the American Dental Association Foundation, nor is it intended to imply that the materials or equipment identified are necessarily the best available for the purpose.
